# Examining the Role of Dyadic Coping on the Marital Adjustment of Couples Undergoing Assisted Reproductive Technology (ART)

**DOI:** 10.3389/fpsyg.2019.00415

**Published:** 2019-03-08

**Authors:** Sara Molgora, Valentina Fenaroli, Chiara Acquati, Arianna De Donno, Maria Pia Baldini, Emanuela Saita

**Affiliations:** ^1^Department of Psychology, Catholic University of Sacred Hearth, Milan, Italy; ^2^Graduate College of Social Work, University of Houston, Houston, TX, United States; ^3^IRCCS Ca 'Granda Foundation Maggiore Policlinico Hospital, Milan, Italy

**Keywords:** dyadic coping, marital adjustment, assisted reproduction (ART), infertile couple, APIM (Actor-Partner Interdependence Model)

## Abstract

A couple is considered to be infertile if unable to conceive after 12 months of unprotected sexual intercourse. An extended body of literature supports that infertility and infertility treatments contribute to emotional, social, sexual, and relational issues that can have a negative impact on each partner's well-being and on the couple relationship. Recent findings suggest that a dyadic approach should be used when working with couples coping with these stressors. However, most research to date has focused on the association between infertility and individual's psychological outcomes, rather than on the experience of infertility-related stress and coping from a relational perspective. Consequently, assuming that infertility is a dyadic stressor and that the ability of the partners to cope with this experience is the result of both individual and relational coping strategies, this study aimed to investigate dyadic coping and marital adjustment among couples at the beginning of an Assisted Reproductive Technology (ART) treatment. A sample of 167 heterosexual couples (*N* = 334) undergoing ART treatment at the fertility clinic of a large hospital in Milan from January to December 2017 was recruited. Each participant completed self-reported questionnaires examining marital adjustment (Dyadic Adjustment Scale) and dyadic coping (Dyadic Coping Questionnaire). Demographics and clinical variables were also collected. Data were analyzed using the Actor Partner Interdependence Model (APIM), testing the effect of each partner's dyadic coping style on their own and their partner's marital adjustment. Results revealed that both women and partners' scores on positive dyadic coping styles (common, emotion-focused, problem-focused, and delegated dyadic coping) contributed to higher marital adjustment. This result suggests that couples unable to engage in this type of reciprocal supportive behaviors and those unsatisfied with their coping efforts may be more vulnerable while undergoing ART treatments. Furthermore, findings highlighted some gender differences for stress communication and negative dyadic coping suggesting the presence of specific dynamics within couples facing an ART treatment. Implications for clinical practice and future research are discussed.

## Introduction

Starting from the historical definition of the World Health Organization that defined infertility as the inability for a couple to conceive after a year of regular, unprotected sexual intercourse (World Health Organization, 1992), its current definition has been expanded to cover a wider spectrum of conditions that affect individuals' and couples' capacity to reproduce (Zegers-Hochschild et al., 2017). In particular, although infertility still represents a disease of the reproductive system—which can be categorized as organic (i.e., linked to organic causes) or functional (i.e., linked to non-organic causes) (Vitale et al., 2017), it is acknowledged that the failure to conceive does not always depend on a disease; thus, the concept of an impairment of function which can lead to a disability has been introduced (Zegers-Hochschild et al., 2017). Worldwide the estimated prevalence of infertility is about 8–12% (Tao et al., 2012), a percentage that increases significantly in Italy, reaching approximately 30% according to data from the Ministry of Health (www.salute.gov.it/portale/fertility). This datum can be explained by considering the phenomenon of the progressive postponement of births in our country, so that, currently, the average age of first childbirth is 32.4 years for women and 35.3 for men, placing Italy as the second country in the European context for delayed maternity (Istat, 2017; Loghi and Crialesi, 2017). This situation supports the relevance of the topic for investigators interested in the study of couples coping with stress in the context of health and family issues (Vitale et al., 2017; Stanhiser and Steiner, 2018).

After a diagnosis of infertility, many couples undergo assisted reproductive technology (ART) treatments in order to become parents. This is a term that includes a wide spectrum of techniques developed to help couples achieve a viable pregnancy. These techniques can be divided in first and second level techniques, with different levels of medicalization. Specifically, for the first levels techniques nowadays couples have the following techniques available: ovulation induction (OI), that involves taking a hormone medication (by tablet or injection) in order to stimulate the production of follicle-stimulating hormone, and artificial insemination (AI) (or intrauterine insemination, IUI), that involves insertion of a male partner's semen through the woman's cervix and into the uterus at or just before the time of ovulation. With reference to the second level techniques, the following procedure are available: *in vitro* fertilization (IVF), that means that the woman's eggs and the man's sperm are left in a culture dish in the laboratory to allow the egg to be fertilized before placing the embryo into the woman's uterus; gamete intrafallopian transfer (GIFT), currently little used, that is considered as a more natural version of IVF because the woman's eggs are retrieved from her ovaries and the egg and sperm are left to fertilize naturally; intracytoplasmic sperm injection (ICSI), that follows the same process as IVF, except involving the direct injection of a single sperm into each egg to achieve fertilization. Furthermore, there are also some procedures that involve the use of donor eggs, donor sperm, or previously frozen embryos. ART treatments have been steadily increasing in recent years (European IVF-monitoring Consortium (EIM) et al., 2017), and, in the same way, the proportion of ART babies among the total number of babies born has increased over the years, now reaching 2.4% (Ferraretti et al., 2017).

Overall, infertility represents a stressful condition, if not a traumatic one, for those who want to have a child because it is associated with the loss and grief connected with not being able to conceive naturally (Koert and Daniluk, 2018). Previous studies reported that the condition of infertility affects the psychological well-being of both women and men, which can feel like depression, guilt, anxiety, and isolation (Schmidt, 2006; El Kissi et al., 2013; Péloquin et al., 2018). If infertility-related distress impacts the quality of life of both partners (Maroufizadeh et al., 2015; Martins et al., 2016), some gender differences are reported (e.g., Ying et al., 2015): infertile women seem to feel more stress about their condition and to experience more depressive symptoms than infertile men (e.g., Berghuis and Stanton, 2002; Kroemeke and Kubicka, 2018). At the same time, gender differences were specifically found also in dealing with ART treatments (Bayley et al., 2009; Davidovà and Pechovà, 2014): indeed, women overall report higher levels of anxiety than men, although over the course of several cycles the average score of anxiety increases for both partners (Schaller et al., 2016). In particular, women's main anxiety seems to be the possible failure to achieve a pregnancy, while men's main anxiety is related with their concern for their partner's health risks (Schaller et al., 2016).

Furthermore, infertile women report lower levels of quality of life than their partners during all phases of the treatment cycle and this difference is greater if the couple has experienced more than one failure of the ART cycles (Agostini et al., 2017). Finally, women are found to implement emotion-focused coping strategies, while men prefer problem-focused coping strategies (Shapiro, 2009). These differences could be partially explained considering that for women the central aspect of infertility is the desire for a child that reinforces their decision to undergo to an ART procedure, while for men the transition to fatherhood often perceived as a more socially defined transition to fulfill the male role (Davidovà and Pechovà, 2014).

Some studies reported that the distress experienced by the partners does not depend on ART techniques (the type of the ART treatment or the number of previous treatments) (Lowyck et al., 2009; Sina et al., 2010; Van Der Merwe and Greeff, 2015); however, according to some authors (Brandes et al., 2009; Gameiro et al., 2012), the distress can impact patients' decisions to discontinue treatment prematurely.

Moreover, distressing feeling and thoughts related to infertility as well as to ART techniques can affect not only each partner—with a specific pattern of adjustment (Moura-Ramos et al., 2016), but also the couple itself as a unit (Cigoli and Scabini, 2006; Schwerdtfeger and Shreffler, 2009; Reis et al., 2013; Turner et al., 2013; Maroufizadeh et al., 2015; Moura-Ramos et al., 2016; Greil et al., 2017). Some authors have analyzed the association between stress related to infertility and ARTs and marital relationship, reporting contrasting results (Van Der Merwe and Greeff, 2015; Chaves et al., 2018). Some authors found that infertility does not reduce marital satisfaction (Amiri et al., 2016), and facing infertility-related stress can contribute to strengthening marital satisfaction and communication among partners, with couples experiencing greater closeness as a consequence of their ability to face the fertility problem as a shared experience (Monga et al., 2004; Schmidt et al., 2005). Others have found that higher levels of stress associated with infertility predicted lower couple satisfaction and an overall worse marital quality (Van Der Merwe and Greeff, 2015; Gana and Jakubowska, 2016). In particular, Van Der Merwe and Greeff (2015) considered four different dimensions of marital quality: quality of communication, intimacy, sexual satisfaction and an overall couple adjustment, and found that the level of infertility-related stress was associated with all four dimensions of marital relationship. This deterioration in the marital relationship following an infertility diagnosis can lead to separation and repartnering (Martins et al., 2014a). Peterson et al. (2003) reported that couples in which men and women perceived similar levels of infertility-related distress reported higher levels of marital adjustment compared with couples in which partners perceived this stress differently. These differences in research findings could be partially explained by considering that several factors (e.g., demographic, economic, social, etc.) may play a role in determining marital satisfaction also in infertile couples (Samadaee-Gelehkolaee et al., 2016). Furthermore, some other variables (e.g., socio-demographic variables, coping strategies, social support, etc.) have been found to mediate the relation between infertility-related stress and the couple relationship, explaining these contrasting results (Ghafouri et al., 2016; Pasha et al., 2017; Greil et al., 2018). Finally, a gender effect for the impact of infertility-related stress on the couple relationship has been found, so that dissimilar results could be partially due to differences between males and females. Indeed, although some authors did not find any difference in marital satisfaction and adjustment between wives and husbands (Yazdani et al., 2016), other authors reported gender differences that move in contrasting directions. For example, Lee and Sun (2000) found that wives were less satisfied than their husbands with their relationship. On the contrary, Peterson et al. (2011) found that a greater percentage of women, compared with men, reported high levels of marital benefit as a positive consequence of the infertility experience. In the same way, differences between males and females emerged when considering the variables that predicted marital satisfaction in infertile couples. For example, Greil et al. (2018) reported that only women, and not men, were significantly more satisfied with their couple relationship when neither partner self-identified as having a fertility problem.

Since infertility represents an unplanned and unexpected stressor, partners usually have considerable difficulty adequately managing this infertility-related stress and activate a variety of coping strategies in order to maintain or regain control over their lives (Peterson et al., 2008). Strategies that partners activate to cope with infertility and following ART can affect both their personal well-being (Rooney and Domar, 2016; Zurlo et al., 2018) as well as their marital well-being (Peterson et al., 2006a, 2008). Several studies have analyzed the coping strategies that partners use to face infertility, distinguishing between more functional or dysfunctional ones (e.g., Bayley et al., 2009). For example, Rockliff et al. (2014) found that the use of escapist coping strategies was associated with increased emotional distress. And, again, Peterson et al. (2006a) reported that avoidance coping strategies are the strongest predictors of decrease in marital adjustment. However, most studies examined these coping strategies from an individual perspective, using the individual as the unit of analysis (Peterson et al., 2008) and did not analyze the reciprocal impact of one partner's coping strategies on his or her partner's well-being or take into account partner interdependence (Pasch and Sullivan, 2017). Using the couple as the unit of analysis, instead, it becomes possible to better investigate the reciprocal influence between partners.

From this dyadic perspective some studies reported gender differences, with men more influenced by their partner than vice versa (Bodenmann et al., 2006). A recent study reported that women's relationship satisfaction more strongly influences their partners' relationship satisfaction (Greil et al., 2018). A similar result was obtained in another study, finding that men's infertility stress was associated with their partners' level of perceived support, but not vice versa (Martins et al., 2014b).

Since infertility can be considered a couple-level (i.e., dyadic) stressor because both partners are affected by this problem and both have to face it, partners are required to cope with these critical experiences together. According to the systemic transactional model of dyadic coping (Bodenmann, 2005; Leuchtmann and Bodenmann, 2018), dyadic coping can be defined as an interpersonal and circular process of managing stressful events shared by both partners within a couple. It is a multidimensional construct depending on several factors (e.g., the situation, individual and dyadic appraisal and goals, partners' competencies), so that partners can engage in positive as well as negative strategies to manage the stressful situation they have to cope with. In particular, different forms of positive dyadic coping can be distinguished: supportive dyadic coping, delegated dyadic coping, and common dyadic coping (Bodenmann, 1995, 2005). Supportive and delegated dyadic coping refer to the efforts of one partner to express solidarity with the other partner (i.e., the stressed partner), providing, respectively, information and practical advice and taking over his or her daily tasks. Specifically, in delegated dyadic coping, the partner is explicitly asked to provide his or her help to the other partner. Both supportive and delegated dyadic coping can be emotion-focused (i.e., focused on partners' emotional distress) or problem-focused (focused on the problem itself). Common dyadic coping refers to the efforts that both partners make together to overcome a direct dyadic stress (Donato et al., 2009). Overall, positive dyadic coping allows partners to maintain or restore their individual well-being as well as to enhance the quality of the couple's relationship, strengthening their sense of we-ness and their reciprocal trust (Bodenmann, 2005; Donato et al., 2009). On the contrary, negative dyadic coping refers to activities following the partner's expression of stress characterized by a negative connotation (e.g., ambivalent or insincere behaviors, superficial interest, hostile comments, etc.). Some studies revealed gender differences in the use of dyadic coping strategies in couples (Staff et al., 2017). For example, women perceive themselves more able to communicate their stress than men (Bodenmann and Cina, 2005; Molgora et al., 2018). Furthermore, women report higher levels of negative dyadic coping (Ledermann et al., 2007), while men perceive dyadic coping to be more efficient than women do (Molgora et al., 2018). Bodenmann et al. (2015) investigated gender differences in support provided to the partner, finding that the support is moderated by the level of stress: in low-stressed conditions men and women provide similar support to the stressed partner, while in a high-stressed situation men provide lower-quality support than women, but only in response to women's emotionally oriented expression of stress.

Over the years, the literature has clearly highlighted how dyadic coping is strongly associated with marital quality, despite cultural and gender differences (Hilpert et al., 2016). Indeed, dyadic coping has been widely investigated in different types of couples and considering several critical events and/or transitions that the couple may face. For example, there are a lot of studies that considered couples facing illnesses or health-related sources of stress (e.g., cancer, cardiac disease, respiratory disease, etc.) (e.g., Hagedoorn et al., 2008; Badr et al., 2010, 2018; Regan et al., 2014; Rottmann et al., 2015; Traa et al., 2015; Switzer et al., 2018; Vilchinsky and Dekel, 2018; Zimmermann and Rauch, 2018). Furthermore, many studies investigated the association between dyadic coping and marital quality in non-clinical couples, in different stage of the life course (late adolescent couples, newly married couples, couples during pregnancy, older couples, etc.) (Landis et al., 2013; Donato et al., 2014; Alves et al., 2018; Breitenstein et al., 2018; Molgora et al., 2018). Specifically, positive dyadic coping was found to predict couple satisfaction and adjustment over time, whereas negative dyadic coping was associated with couple distress (Bodenmann et al., 2006; Falconier et al., 2015; Rusu et al., 2018). Furthermore, the association between dyadic coping and marital quality can be mediated by the partners' ability to communicate their stress (Ledermann et al., 2010). However, to the authors' knowledge, only one other recent study (Chaves et al., 2018) has specifically analyzed the relationship between dyadic coping and marital adjustment in relation to infertility, and in particular in couples undergoing ART, highlighting the central role of men's dyadic coping strategies for the marital adjustment of both partners: indeed, while males' marital adjustment is influenced by the perception of their own coping, females' marital adjustment is influenced by their partners' perception. However, this study, although it considered both women and men, did not use a properly dyadic approach to investigate the reciprocal influence between partners.

The present study is aimed at investigating the differences between men's and women's perception of dyadic coping strategies as well as marital adjustment in a sample of Italian couples undergoing ART. Furthermore, we tested the relationship between dyadic coping and marital adjustment. Because of the shared nature of the infertility experience, we used the Actor-Partner Interdependence Model (APIM) to analyze the effect of each partner's perception of dyadic coping strategies on its own and partners' marital adjustment. Since, to our knowledge, no previous study has investigated the association between dyadic coping and marital adjustment in infertile couples using this methodology, we adopted an explorative approach, testing the relation between all the different dyadic coping strategies and marital adjustment. However, following the results of a previous study reporting a stronger association between positive dyadic coping and marital adjustment (Falconier et al., 2015), and considering that the infertility experience has a common (i.e., dyadic) dimension, beyond individual gender-related specificities, we will expect that positive dyadic coping strategies, and in particular common dyadic coping, would have been associated with higher individual's (i.e., actor effect) and partner's (i.e., partner effect) perceptions of marital adjustment.

## Methods

### Participants

From January to December 2017, a total of 230 couples, which represent all the couples entering an ART program at a public hospital in Milan, were contacted regarding participation in this study. Of these, 30 did not consent to participate, while 200 agreed to take part in this cross-sectional study. Of this number, 33 couples were excluded because of incomplete questionnaires, with the final sample comprised of 167 childless couples. Chi-square and independent samples *t*-test analyses showed no differences between couples who completed the questionnaires and those who did not complete all measures as regards socio-demographic and infertility-related variables as well as the other study variables (dyadic coping and couple adjustment).

Mean age of participants was 36.13 (*SD* = 3.92; range = 22–44) for women and 38.9 (DS = 5.08; range: 22–58) for men. 35.8% of women and 28.3% of men in the sample had a high-school diploma; 15.1% of women and 11.0% of men had a junior high-school diploma; 33.5% of women and 37.6% of men were college graduates. Almost all the participants (91.1% of women and 96% of men) were employed. Most of the women (59%) are office workers; among men, 18.6% are blue collar workers, 35.9% are office workers, and 13.9% are managers.

As for characteristics related to infertility, 45.1% of diagnoses were female factor, 27.8% male factor, 11.3% both partners' factor; 15.8% of infertility diagnoses were idiopathic. More than half of the couples (57.1%) never underwent an ART cycle; 42.9% had already undergone an average of 2 (mean = 2.29; *SD* = 1.58) ART treatment. In preparation for our analysis, couples who had completed previous ART attempts were compared to couples with no previous history of ART. No differences were found regarding the study variables of interest for women. On the contrary, men who had undergone previous ART reported lower levels of dyadic coping [*F*_(1, 165)_ = 5.31; *p* < 0.05] and marital adjustment [*F*_(1, 165)_ = 4.83; *p* < 0.05] than men who had never undergone treatments. 64.5% of couples were enrolled in IVF and 32% in ICSI treatments.

### Measures

#### Dyadic Adjustment Scale (DAS) (Spanier, 1976; Gentili et al., 2002)

This scale measures couple's adjustment through 32 items: 31 items are related to specific aspects of a couple's interactions and one item assesses the overall happiness with the relationship. The higher the total score, obtained by summing the 32 items, the greater is the perceived couple adjustment. The instrument shows good internal consistency both for men (Cronbach's alpha = 0.90) and women (Cronbach's alpha = 0.90).

#### Dyadic Coping Questionnaire (DCQ)^1^ (Bodenmann, 2000; Donato et al., 2009)

This scale measures dyadic coping behaviors through 41 items on a Likert-type 5-point scale ranging from “never” to “very often.” In particular, 39 items are related to six different styles of dyadic coping (stress communication−8 items, emotion-focused−6 items, problem-focused−4 items, delegated−4 items, negative−10 items, and common−7 items) while the last two items evaluate satisfaction and efficacy for dyadic coping. The scores of the negative coping items have to be reversed, so the higher the score of this subscale, the lower is the use of hostile, ambivalent or superficial strategies by the partner. The mean of all positive and reversely coded negative items reveals the partners' perceived total dyadic coping skills. The higher the score, the more the partners feel they are jointly managing the stressful situation. The subscales related to stress communication, emotion-focused, problem-focused, delegated and negative dyadic coping consider both self-perception (i.e., one's own dyadic coping) and other perception (i.e., partner's dyadic coping). Considering both self- and other-perception allows a comparison between partners' perceptions and an investigation of the balance between the support provided and received by partners, both at an intra-individual and an interpersonal level (Donato et al., 2009). For this reason, we analyzed both self-perception and other perception in our models. Examples of items are the following: “I tell my partner openly how I feel and that I would appreciate his/her emotional support” (stress communication, self-perception); “My partner shows me that he/she is stressed and is not feeling well” (stress communication, other-perception); “I listen to my partner, give him/her the opportunity to express his/her stress, comfort and encourage him/her” (emotion-focused, self-perception); “My partner listens to me, gives me the opportunity to express my stress, comforts and encourages me” (emotion-focused, other-perception); “I try to analyze the situation together with my partner and help him/her to understand and deal with the problem” (problem-focused, self-perception); “My partner helps me to see the stressful situation in a different light and to put the problem in perspective” (problem-focused, other-perception); “I take on things that my partner would normally do in order to help him/her out” (delegated, self-perception); “When I am too busy, my partner helps me out” (delegated, other-perception); “When my partner is stressed, I withdraw from him/her” (negative, self-perception); “My partner makes fun of my stress and mocks me” (negative, other-perception); “We help each other to put the problem in perspective and see it in a new light” (common). The reliability of each subscale and total score on dyadic coping was satisfactory, with scores ranging from 0.78 to 0.90 for men and from 0.74 to 0.93 for women.

A socio-demographic and clinical form was included, including information about age, educational level, job situation and clinical variables about infertility, i.e., the diagnosis (when known), the number of previous ART treatments and the type of ART treatment couples are undergoing.

### Procedure

The research project was approved by the Institutional Review Board of the Catholic University of the Sacred Heart. All participants were informed about the research aim and methodology and signed a written informed consent form. Data were collected at the beginning of the assisted reproductive technology procedure. Specifically, both partners were recruited when they were in a day hospital for some preliminary exams before they entering treatment (e.g., hormonal stimulation). The beginning of treatment took place few days later they have completed the questionnaire. Each partner was asked to complete an on-site questionnaire independently from the other partner. Anonymity and data confidentiality were guaranteed.

### Data Analysis

Descriptive statistics were conducted to illustrate the sample characteristics for demographics, clinical factors, and variables of interest. Differences between the men and women on dyadic coping and couple adjustment were investigated with paired-samples *t*-test, by comparing the score of the two partners on each of the Dyadic Coping Questionnaire subscale and on the total score of the Dyadic Adjustment Scale. Pearson *r* correlations were used to assess the association between dyadic coping and dyadic adjustment.

**Figure 1:**
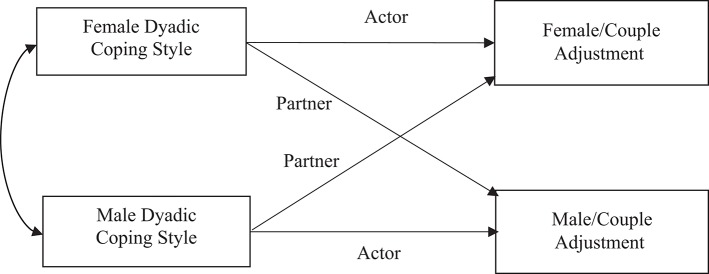
Actor and partner effects of dyadic coping predicting couple adjustment using the APIM model.

To determine the impact of women and men's dyadic coping on their own as well as their partners' scores on marital adjustment, data were analyzed with the Actor Partner Interdependence Model (APIM), because this model accounts for the non-independence of dyadic data and because this approach treats data from each member of the dyad as nested within the same group (Kenny et al., 2006). The APIM model has been extensively used in the study of close relationships, attachment, caregiving, and couples coping with stress and allows the investigators to consider the reciprocal influence of each partner on their own and their partner's outcome measure simultaneously (see Figure 1). The model states that the person's score on an independent variable can influence their own, as well as their partner's score, on the dependent variable. For this study, the actor effect was the impact of a person's dyadic coping on his or her own marital adjustment. The partner effect was the impact of each person's dyadic coping on the marital adjustment of the other member of the dyad. In the present study, we examined couple adjustment using both actor and partner dyadic coping strategies scores—both self-perceived and other perceived—as predictor variables. Women and men mean-centered predictor variables were regressed on their outcome variables in a single regression model. We also investigated gender interactions to test whether gender differences were present; in case of a significant actor or partner effect interaction, separate regression analyses for females and males were conducted. Within our results, standardized coefficients indicate that an increase in the predictor variable resulted in an increase of the dependent variable. To conduct dyadic data analysis on the present database, the Intra Class Correlation Coefficient (ICC) was calculated between the outcome variables of women and men to examine the amount of non-independence within the couple. Then, an Onmibus Test of Distinguishability was conducted to assess whether treating the dyad as distinguishable improved the fit of the model. We then tested whether gender acted as a moderator of actor and/or partner effects. Hence, an interaction model using REML estimation was tested first, followed by a two-intercept approach. If no significant interaction was found between role and actor or partner effect, the standardized coefficient of the average effect is reported to remind the reader of its significance. Analyses were completed with SPSS package, version 24, using the mixed models procedure, with an alpha level of 0.05.

## Results

### Descriptive Information of the Variables of Interest

Table 1 provides an overview of the study's variables of interest, with reported means and variance indicators of dyadic coping (total score and subscales) and marital adjustment.

**Table 1:** Descriptive statistics of relationship satisfaction and dyadic coping scores for men and women.

	**Women Mean (*****SD*****)**		**Men Mean (*****SD*****)**	
DAS total score	124.37 (12.25)		125.59 (11.73)	
	**Self-perception**	**Other-perception**	**Self-perception**	**Other-perception**
DCQ total score	120.79 (16.48)		117.40 (16.13)	
Stress communication	10.77 (2.71)^***^	8.60 (2.64)	9.45 (2.90)	11.14 (3.04)^***^
Emotion-focused supportive DC	9.78 (1.73)	9.50 (2.12)	9.56 (1.88)	9.78 (2.20)
Problem-focused supportive DC	5.88 (1.43)	6.05 (1.40)	5.90 (1.49)	5.90 (1.59)
Delegated DC	5.24 (1.34)	5.10 (1.73)	5.52 (1.49)	5.15 (1.60)
Negative DC	11.71 (1.75)	17.41 (3.17)	11.36 (1.83)	17.60 (3.54)
Common DC	21.92 (4.12)		22.55 (8.38)	
DC satisfaction	3.30 (0.73		3.23 (0.84)	
DC efficacy	2.34 (0.67)		4.31 (0.74)^***^	

*Statistically significant values are shown in bold*.

*** *p < 0.001*.

Table 2 presents the bivariate associations between dyadic coping and marital adjustment for the two genders.

**Table 2:** Bivariate correlations between relationship adjustment and dyadic coping scores for men and women.

**Variables**	**1**	**2**	**3**	**4**	**5**	**6**	**7**	**8**	**9**	**10**	**11**	**12**	**13**	**14**
Total Score DAS	0.45^***^	0.22^**^	0.73^**^	0.57^***^	0.27^***^	0.21^**^	0.41^*^	0.23^***^	0.55^**^	0.34^***^	0.35^***^	0.33^***^	0.53^***^	0.49^***^
Stress communication_SP	0.37^***^	0.35^***^	0.31^***^	0.27^***^	0.24^**^	0.20^**^	0.06	0.29^***^	0.29^***^	0.33^***^	0.39^***^	0.03	0.23^**^	0.12
Emotion-focused_SP	0.42^***^	0.41^***^	0.29^***^	0.58^***^	0.38^***^	0.27^***^	0.31^***^	0.35^***^	0.59^***^	0.46^***^	0.41^***^	0.26^**^	0.67^***^	0.54^***^
Problem-focused_SP	0.32^***^	0.27^**^	0.51^***^	0.20^**^	0.57^***^	0.41^***^	0.39^***^	0.49^***^	0.42^***^	0.24^**^	0.30^***^	0.42^***^	0.45^***^	0.28^***^
Delegated_SP	0.23^**^	0.11	0.33^***^	0.28^***^	0.07	0.36^***^	0.24^**^	0.34^***^	0.26^**^	0.14	0.18^*^	0.22^**^	0.25^**^	0.09
Negative_SP	0.28^**^	0.15^*^	0.38^***^	0.09	0.04	0.61^***^	0.08	0.43^***^	0.19^*^	0.08	0.22^**^	0.07	0.19^*^	0.09
Stress communication_OP	0.40^***^	0.30^***^	0.32^***^	0.43^***^	0.32^***^	0.08	0.06	0.07	0.39^***^	0.26^**^	0.14	0.65^***^	0.25^**^	0.168^*^
Emotion-focused_OP	0.52^***^	0.46^***^	0.63^***^	0.39^***^	0.21^**^	0.29^**^	0.34^***^	0.47^***^	0.21^**^	0.22^**^	0.24^**^	0.14	0.23^**^	0.14
Problem-focused_OP	0.33^***^	0.35^***^	0.42^***^	0.33^***^	0.13	0.18^*^	0.14	0.48^***^	0.19^*^	0.62^***^	0.54^***^	0.42^***^	0.58^***^	0.53^***^
Delegated_OP	0.29^***^	0.42^***^	0.40^***^	0.28^***^	0.24^**^	0.17^*^	0.26^**^	0.55^***^	0.37^***^	0.22^**^	0.55^***^	0.22^**^	0.39^***^	0.37^***^
Negative_OP	0.31^***^	0.19^*^	0.45^***^	0.18^*^	0.16^*^	0.73^***^	0.17^*^	0.38^***^	0.25^**^	0.27^***^	0.33^***^	0.15	0.44^***^	0.36^***^
Common	0.68^***^	0.47^***^	0.60^***^	0.39^**^	0.24^**^	0.26^**^	0.36^***^	0.61^***^	0.50^***^	0.40^***^	0.33^***^	0.52^***^	0.22^**^	0.24^**^
Satisfaction	0.65^***^	0.40^***^	0.54^***^	0.33^***^	0.16^*^	0.24^**^	0.28^***^	0.60^***^	0.45^***^	0.43^***^	0.33^***^	0.72^***^	0.43^***^	0.66^***^
Efficacy	0.55^***^	0.32^***^	0.47^***^	0.36^***^	0.14	0.21^**^	0.28^***^	0.58^***^	0.37^***^	0.43^***^	0.28^***^	0.70^***^	0.84^***^	0.28^***^

* *p < 0.05*;

** *p < 0.01*;

*** *p < 0.001*.

*SP, Self-Perceived; OP, Other-Perceived*.

*Men correlations are reported above the diagonal, women scores are reported below*.

### Differences Between Men and Women

Paired-sample *t*-test analyses showed no differences between men and women either regarding the DAS score or the DCQ score, with the exception of stress communication and efficacy of dyadic coping. Women reported significantly higher stress communication scores, [*t*_(166)_ = 4.270; *p* < 0.001) and were perceived as more able to communicate their stress to [*t*_(166) =_ −8.115; *p* < 0.001). Moreover, men reported significantly higher efficacy related to dyadic coping than women [*t*_(159)_ = −32.674; *p* < 0.001].

### APIM of Dyadic Coping Strategies on Couple's Adjustment

The analysis examined actor and partner effects of each dimension of dyadic coping included in the Dyadic Coping Questionnaire (both self-perceived and other-perceived) in predicting marital adjustment among the dyads involved in the current study.

First, *self-perceived* dyadic coping strategies were examined, with separate analysis conducted for each subscale (Table 3).

**Table 3:** Predicting self-reported marital adjustment from *self-perceived* dyadic coping scores.

		**95% confidence interval**	
	**β**	**Lower**	**Upper**
**PREDICTING** ***FEMALE*** **MARITAL ADJUSTMENT**			
**Own marital adjustment (actor effects)**			
F Stress Communication	3.82^***^	1.79	5.84
F Emotion-Focused DC	4.39^***^	2.59	6.18
F Problem-Focused DC	2.94^***^	1.77	4.12
F Delegated DC	2.37^*^	0.44	4.30
F Negative DC	3.17^**^	0.86	5.49
**Partner's marital adjustment (partner effects)**			
M Stress Communication	2.00^**^	0.79	3.21
M Emotion-Focused DC	3.23^***^	1.57	4.89
M Problem-Focused DC	2.36^***^	1.21	3.53
M Delegated DC	1.95^*^	0.18	3.72
M Negative DC	0.94	−1.29	3.18
**PREDICTING** ***MALE*** **MARITAL ADJUSTMENT**			
**Own marital adjustment (actor effects)**			
M Stress Communication	0.72	−1.08	2.53
M Emotion-Focused DC	5.40^***^	3.91	6.88
M Problem-Focused DC	2.94^***^	1.77	4.12
M Delegated DC	1.96^*^	0.26	3.66
M Negative DC	3.78^***^	1.73	5.83
**Partner's marital adjustment (partner effects)**			
F Stress Communication	2.00^**^	0.79	3.21
F Emotion-Focused DC	2.83^**^	1.22	4.45
F Problem-Focused DC	2.36^***^	1.20	3.53
F Delegated DC	2.06^*^	0.21	3.92
F Negative DC	0.91	−1.19	3.02

* *p < 0.05*;

** *p < 0.01*;

*** *p < 0.001*.

For *self-perceived stress communication* overall actor and partner effects on marital adjustment were detected: both men and women reporting high levels of stress communication were more likely to report high scores on couples' adjustment. Furthermore, individuals whose partners reported high scores on stress communication were also predicted to report high scores on couple's marital adjustment scores. Gender was a significant moderator of the actor effect (*p* < 0.05), while the interaction between gender and partner effect approached significance (*p* = 0.06). Thus, after calculating simple slopes, our results indicated that only the female actor effect was statistically significant (*p* < 0.001). For *self-perceived emotion-focused, problem-focused* and *delegated* dyadic coping, each analysis revealed both actor and partner effects of the dyadic coping strategy on the score reported on the DAS. Individuals reporting high levels of these coping strategies were more likely to report higher marital adjustment scores. Similarly, men and women whose partner reported higher levels of these dyadic coping strategies were predicted to report high levels of couple adjustment. In our analysis about *self-perceived negative* dyadic coping, there is evidence of only an actor effect of negative dyadic coping on couple's adjustment: individuals reporting high levels of this coping strategy were more likely to report higher scores on couple's adjustment.

*Other-perceived* subscales were then examined (Table 4). Actor and partner effects were identified for all the subscales investigated: higher marital adjustment scores were predicted for those who scored high on all these dyadic coping dimensions as well as for participants whose partners had elevated scores. Specifically, for *other-perceived stress communication, emotion-focused* dyadic coping, *problem-focused* dyadic coping, and *delegated* dyadic coping, higher scores on the outcome measure were predicted for men and women who scored high on these subscales, and high marital adjustment was predicted also for those whose partner presented elevated scores on these coping behaviors. Additionally, no mean level differences in couple functioning score were identified for the two partners. For *other-perceived negative* dyadic coping, gender was a significant moderator of both actor and partner effects. Results indicate that only the partner effect for the score reported by men was significant (*p* < 0.01), while both male and female actor effects were statistically significant.

**Table 4:** Predicting self-reported marital adjustment from *other-perceived* dyadic coping scores.

		**95% confidence interval**	
	**β**	**Lower**	**Upper**
**PREDICTING** ***FEMALE*** **MARITAL ADJUSTMENT**			
**Own marital adjustment (actor effects)**			
F Stress Communication	3.42^***^	2.08	4.75
F Emotion-Focused DC	4.57^***^	2.71	6.43
F Problem-Focused DC	3.03^**^	1.08	4.98
F Delegated DC	3.03^**^	1.29	4.77
F Negative DC	4.74^***^	2.83	6.64
**Partner's marital adjustment (partner effects)**			
M Stress Communication	2.39^***^	1.07	3.73
M Emotion-Focused DC	2.85^**^	1.04	4.65
M Problem-Focused DC	2.00^*^	0.29	3.71
M Delegated DC	2.18^*^	0.27	4.09
M Negative DC	−0.96	−2.71	0.79
**PREDICTING** ***MALE*** **MARITAL ADJUSTMENT**			
**Own marital adjustment (actor effects)**			
M Stress Communication	3.42^***^	2.08	4.75
M Emotion-Focused DC	4.69^***^	3.01	6.36
M Problem-Focused DC	2.82^**^	1.25	4.40
M Delegated DC	4.15^***^	2.38	5.92
M Negative DC	1.68^*^	0.02	3.36
**Partner's marital adjustment (partner effects)**			
F Stress Communication	2.39^***^	1.07	3.73
F Emotion-Focused DC	2.94^**^	1.21	4.67
F Problem-Focused DC	3.72^***^	1.92	5.52
F Delegated DC	2.14^**^	0.53	3.75
F Negative DC	3.22^**^	1.41	5.05

* *p < 0.05*;

** *p < 0.01*;

*** *p < 0.001*.

Finally, we examined the influence of *common* dyadic coping*, satisfaction and efficacy* of couples' dyadic coping behaviors in predicting the couples' adjustment (Table 5). From our analysis, there is evidence that gender was a significant moderator of both actor and partner effects. Only the female actor effect was significant (*p* < 0.001), while both male and female partner effects were statistically significant (*p* < 0.001). Finally, both actor and partner effects of satisfaction with dyadic coping behaviors existed in our sample (*p* < 0.001). Gender moderated the actor effect (*p* < 0.05), which was significant for both men and women (*p* < 0.001) and greater for women. Finally, mean-level differences were identified for efficacy of dyadic coping between the two partners (*p* < 0.05), with greater scores on marital adjustment reported by women. Actor (*p* < 0.001) and partner effects (*p* < 0.001) were identified.

**Table 5:** Predicting self-reported marital adjustment from *common* dyadic coping, *satisfaction* with dyadic coping behaviors and *efficacy* of dyadic coping behaviors.

		**95% confidence interval**	
	**β**	**Lower**	**Upper**
**PREDICTING** ***FEMALE*** **MARITAL ADJUSTMENT**			
**Own marital adjustment (actor effects)**			
F Common DC	12.35^***^	10.03	14.67
F Satisfaction with DC	7.03^***^	5.36	8.69
F Efficacy of DC	6.94^***^	4.85	9.02
**Partner's marital adjustment (partner effects)**			
M Common DC	1.27^*^	0.14	2.41
M Satisfaction with DC	2.72^***^	1.26	4.17
M Efficacy of DC	2.30^*^	0.43	4.16
**PREDICTING** ***MALE*** **MARITAL ADJUSTMENT**			
**Own marital adjustment (actor effects)**			
M Common DC	0.06	−0.58	1.85
M Satisfaction with DC	4.45^***^	2.99	5.92
M Efficacy of DC	5.25^***^	3.37	7.13
**Partner's marital adjustment (partner effects)**			
F Common DC	9.77^***^	7.28	12.27
F Satisfaction with DC	3.84^***^	2.16	5.51
F Efficacy of DC	2.82^**^	0.84	4.81

* *p < 0.05*;

** *p < 0.01*;

*** *p < 0.001*.

## Discussion

### Gender Differences in Dyadic Coping and Marital Adjustment

Since infertility and ART treatments represent stressful experiences for each partner as well as for the couple relationship, independently of the specific type of treatment (Van Der Merwe and Greeff, 2015; Koert and Daniluk, 2018), the present study examined the relationship between dyadic coping and marital adjustment in a sample of Italian infertile couples at the beginning of ART treatment. In particular, we explored whether specific dyadic coping strategies, both self-perceived and other-perceived, have an impact on marital adjustment, considering direct (actor) as well as indirect (partner) effect. Furthermore, we were interested in exploring gender differences, since the literature highlights the presence of differences between males and females both for coping strategies as well as for the impact that infertility has on individual and relational well-being (Ying et al., 2015; Staff et al., 2017).

An initial result is that partners overall have similar representations of their relationship quality in terms of adjustment as well as of their ability to jointly cope with the critical experience of ART. These findings are partially congruent with previous studies on couples' adjustment in non-clinical samples, indicating no significant gender difference (Gager and Sanchez, 2003; Jackson et al., 2014), although other studies have found wives generally reporting a lower marital adjustment than husbands (Amato et al., 2007; Kamp Dush et al., 2008). Considering the specific condition of infertility, the findings reported in literature are controversial: indeed, while some authors did not find any significant difference in marital adjustment between infertile women and infertile men (Yazdani et al., 2016), other researchers found gender differences going in contrasting directions (Lee and Sun, 2000; Peterson et al., 2011). Hence, it is possible to assert that the difference between wives and husbands as regards marital adjustment is partially connected to the different strategies female and male partners adopt in order to cope with the infertility experience and, therefore, to the different impact that infertility has on the individual well-being of women and men. Indeed, although some studies underscored that the infertility condition affects the psychological well-being of both women and men (Schmidt, 2006; El Kissi et al., 2013; Péloquin et al., 2018), the majority of studies today are in agreement that the infertility experience is more stressful for women than for men, with infertile women generally reporting more stress regarding their condition (Berghuis and Stanton, 2002; Peterson et al., 2008; Ying et al., 2015; Greil et al., 2018; Kroemeke and Kubicka, 2018). At the same time, according to researchers who do not find differences between the partners, it is possible that the stress and emotional hardship connected to the experience of infertility are shared by the partners through a spill-over effect from one partner to the other, and that, for this reason, couple adjustment also presents similar levels. It should also be emphasized that, according to some authors, the congruence between the partners as regards couple adjustment representation is a positive factor for the couple itself and a protective factor, both in infertile couples as well as in the context of dyadic coping (Peterson et al., 2003; Iafrate et al., 2012).

Since dyadic coping impacts on marital adjustment, as several studies have extensively reported (e.g., Bodenmann et al., 2006; Falconier et al., 2015; Rusu et al., 2018), we can suppose that the presence in our study of similar levels of marital adjustment in males and females may be explained considering the specific role of coping strategies and the several effects that these strategies have not only at an intra-individual level, but also at interpersonal level (Donato et al., 2009). Indeed, dyadic coping strategies, that represent the efforts of both partners to face together with a critical and stressful experience, can contribute to a greater sharing of the feelings and thoughts related to infertility and, in this way, to a perceived more similar distress related to this experience.

In our study, significant differences existed between women and their male partners only for stress communication, both when self- and other-perceived communication were assessed, as well as efficacy. Specifically, women perceived themselves more able to communicate stress to their partner than men and, at the same time, men perceived their partner more able to communicate stress than themselves. Overall, this finding confirms previous studies that reported some gender specific patterns within the couple relationship (Helgeson, 2011) and, specifically, the greater ability of women to communicate their distress to the partner and the greater use of avoidant behaviors among men, which could be related to a major difficulty in expressing and communicating their emotions (Jackson et al., 2014). Furthermore, it is coherent with other studies that highlight how women's infertility stress is greater than that of men, despite being related to partner behaviors (Martins et al., 2014b). Moreover, our result confirms other studies on dyadic coping that revealed gender differences in the use of dyadic coping strategies in couples (Staff et al., 2017), with women perceiving themselves more able to communicate their stress than men (Bodenmann and Cina, 2005; Molgora et al., 2018). We can hypothesize that, even in our sample, gender differences in communication, now widely recognized in the literature, become evident in a situation–the ART experience–that sees women particularly involved both on the physical level (think of all the exams to which women are subjected, to the hormonal stimulation, to the pick-up to which they are prepared, etc.) and the psychological one. Indeed, it is likely that women experience a very intense stress, higher than what men experience in the same moment, but that they also can better express and communicate it to the partner. Finally, this result highlights again a perceptual congruence between the two partners within the couple (Iafrate et al., 2012); this congruence could be connected to the overall good couple relationship found in our sample. Indeed, although the Dyadic Adjustment Scale did not have cut-off values, the mean of our samples denotes high scores, in line with normative data on the general population, indicating a satisfactory couple relationship. From a clinical perspective, we could wonder whether this good quality represents a defense on the part of the partners who are facing the pain of infertility. Certainly, we can argue that in this specific moment the couple seems to be working to cope with some individual struggles connected to this experience which have some gender-specific dimensions.

As for efficacy, men perceived their dyadic coping to be more efficient than women, confirming findings of a previous study on couple transitioning to parenthood in which men were found to perceive dyadic coping too be more efficient than women did (Molgora et al., 2018). We can speculate that this perception can be associated with differential stress-related consequences of the infertility as well as the ART experience between men and women (Bayley et al., 2009; Davidovà and Pechovà, 2014; Ying et al., 2015).

Considering the association between dyadic coping and marital adjustment, as the literature has widely recognized on non-clinical couples in different stages of the life course (Donato et al., 2014; Falconier et al., 2015; Molgora et al., 2018), results confirm that in our sample of infertile couples undergoing an ART treatment the ability to manage the infertility-related stress as a “common problem” is linked to a better couple adjustment and increases marital adjustment. Although some studies have reported that men and women manage the stress for infertility by activating gender-specific individual coping strategies (Peterson et al., 2006b), our findings show that partners are able to contemporaneously face this critical experience together, living their problem as a “dyadic problem.”

### Association Between Dyadic Coping and Marital Adjustment

The use of the APIM promoted a meaningful examination of the association between dyadic coping and marital adjustment, and of the experience of infertile couples as they approach assisted reproduction. As outlined by the theoretical framework and the available evidence to date, some positive dyadic coping dimensions were found to be associated with higher levels of relationship adjustment both in women and their partners, without gender differences. We might suppose that these findings explain the common dimension of the infertility experience, which, as we reported in the introduction, involved both partner beyond the type of diagnosis and requires the activation of functional coping strategies both in women and in their partners. In particular, both an actor and a partner effect were found for emotion-focused, problem-focused and delegated dyadic coping, both self-perceived and other-perceived, as well as for common coping, meaning that women and their partners reporting high levels of these strategies were more likely to report higher adjustment scores and, at the same time, men and women whose partners reported higher levels of these dyadic coping strategies were predicted to report high levels of couple adjustment. These results are consistent with the literature on dyadic coping, as more effective coping styles have been associated with better relational outcomes across samples and over time (Bodenmann and Cina, 2005; Falconier et al., 2015; Bodenmann et al., 2016).

Although for most of the coping subscales actor and/or partner effects were similar in both men and women, in some specific dimensions gender specificities emerged. Specifically, we found only a female actor effect of stress communication (self-perceived) on couple adjustment, partially supporting what has been already found in studies showing that women assign greater importance than men to communication within the couple (Matud, 2004).

Moreover, although for both partners the actor effect of dyadic coping satisfaction on couple adjustment is significant, the results show that this effect is stronger for women. Therefore, although for both partners the satisfaction of being able to face a stressful event together contributes to their marital relationship, we can assume that this connection acquires a specific centrality for the woman, more directly involved in the ART process than the partner and more sensitive to the partner's dyadic support. At this crucial moment in a couple's life, it is possible that the woman's marital adjustment is more strongly linked to the feeling that the partner is engaged in these “common” efforts.

Some gender specificities have also been found for men. Specifically, the results show a stronger partner effect for men in common dyadic coping. In our study men's perception of couple adjustment is predicted by the partners' common dyadic coping perception. As already mentioned above, the ART process mainly involves the woman, who is subjected to a great physical and mental stress. We can hypothesize that in this phase men are particularly focused on the well-being of their partner and that their couple's adjustment is strongly influenced by the woman's perception that the dyad is coordinating their strategies. At the same time, the fact that women feel they are jointly coping with the ART process can make the partner feel more involved, and therefore more satisfied with the couple relationship. Moreover, other studies using this dyadic approach reported an overall gender difference, with men more influenced by their partner than vice versa (Bodenmann et al., 2006; Greil et al., 2018).

Finally, the findings about negative coping indicate that men are more satisfied with their relationship if their partners feel empathetically (without superficial or ambivalent behaviors) supported by them. Once again, we might suppose that the male perception of the relationship is in this phase very focused on the needs of the woman, who is especially involved in the process of ART, not only at a psychological level but also at a physical one.

Although our study was among the first to examine dyadic coping in the context of infertility, results confirmed what the literature has presented in other groups of couples facing other stressors: a relationship exists between dyadic coping and marital adjustment. The partners' ability to jointly cope with the stressful ART process makes the couple more adjusted. The results also show how these effects are reciprocal within the couple: one's partner's perception about dyadic coping affects one's own couple adjustment, and vice versa. However, some gender specificities emerged: women and men show some typical differences in their relationship and in the way they cope with stress. Women are strongly involved in the ART process, and this can activate some specific couple dynamics, with a particular focus on the needs of the woman.

In conclusion, the intrinsically dyadic nature of the infertility experience can contribute to explaining the presence of a high congruence in our sample between men and women with respect to the dyadic coping strategies put into action and to the impact that these strategies have on marital adjustment, as well as to the effects of reciprocal influence between one's perception and that of one's partner (cross-partner effects). Nevertheless, some specific aspects emerge connected to gender that are expressed in differences in some dimensions of dyadic coping, and which in part reflect differences also found in the general population, which speak to a gender specific functioning. These differences can also be connected to specific thoughts and feelings linked to this experience that see men and women involved in different ways. The differing involvement of men and women in the infertility experience and subsequent treatments can be understood in terms of a socio-cultural dimension, that is connected to the different meanings and expectations that society nurtures with respect to motherhood and fatherhood, as well as in terms of more structural aspects, which we could say pertain to identity and are connected to the deep significance that motherhood has for women, as distinct from what fatherhood means for men.

### Limitations

Some limitations affect this study. First, some medical variables related to infertility (i.e., partner with the diagnosis of infertility, number of treatment, type of ART) were not controlled. If for methodological reasons mostly connected to the sample size, we did not include in our models the medical variables in our possession (in particular, the number of IVF treatments), we can assume that the actor and partner effects of dyadic coping strategies on marital adjustment are impacted by these variables. Furthermore, this is a cross-sectional study that involves couples undergoing ART, so the direction of the association we tested is theory driven. A longitudinal design is needed to better understand the association between dyadic coping and marital adjustment and to find trajectories of change over time. Finally, we have considered the impact of dyadic coping styles on marital adjustment. Future research could investigate the role of individual well-being (e.g., anxiety and depressive symptoms) as a moderator of this relation. Indeed, some studies have reported how dyadic coping impacts individual mental health (e.g., Bodenmann et al., 2008).

Despite these limitations, the present study underscored the importance of considering the decision to undergo assisted reproductive technology treatments as a shared experience, i.e., a dyadic stressor, which requires dyadic strategies.

### Clinical Implications

These research findings have important clinical implications and may assist in developing interventions to promote individual and dyadic coping with infertility. The presence of good levels of couple adjustment, similar between partners, and the fact that adaptive coping strategies impact on couple adjustment, suggest that in this crucial moment (start of an ART procedure), the couple can represent an important resource for partners. This could be a useful element also for medical staff (doctors, nurses, etc.), who can rely on a good “couple's alliance” managing the stressful ART process. From a clinical point of view, the results obtained lead us to think that the couple could be positively considered during the ART process as a useful “common container” of the individual efforts, often connected to gender-specific components, of each partner in the ART process. In other words, the results lead us to ask ourselves if the couple relationship could represent not the specific focus (i.e., “the object”) of psychological interventions, but rather an effective resource through which to enhance the well-being of the individual partners, so differently involved in the ART process. Indeed, in this phase of life partners show to be, for different reasons, perhaps also defensively, satisfied with their relationship and able to jointly manage the common ART experience.

## Author Contributions

SM contributed to developing the study design and to writing the entire manuscript. VF contributed to developing the study design, the data set for individual, and dyadic data analysis, and to writing the method, and discussion sections. CA performed dyadic data analysis and contributed to writing the method, results and discussion sections. ADD contributed to developing the study design and to reviewing the manuscript. MB contributed to the data collection. ES contributed to developing the study design, and to writing the discussion section. All the authors reviewed and approved the manuscript for publication.

### Conflict of Interest Statement

The authors declare that the research was conducted in the absence of any commercial or financial relationships that could be construed as a potential conflict of interest.

## Footnotes

^1^DCQ is the only scale on dyadic coping validated for the Italian population (Donato et al., 2009). It is somewhat longer than the more recent Dyadic Coping Inventory (DCI; Bodenmann, 2008) that measures dyadic coping behaviors through 37 items. DCQ and DCI measure the same subscales and consider both self-perception (coping by oneself) and other perception (coping by partner). However, the DCQ includes two single items, respectively, for dyadic coping satisfaction and efficacy, while the DCI has only one item for the perceived quality of dyadic coping.

## References

[B1] Agostini F. Monti F. Andrei F. Paterlini M. Palomba S. La Sala G. B. (2017). Assisted reproductive technology treatments and quality of life: a longitudinal study among subfertile women and men. J. Assist. Reprod. Genet. 34, 1307–1315. 10.1007/s10815-017-1000-9 28733802 PMC5633563

[B2] Alves S. Fonseca A. Canavarro M. C. Pereira M. (2018). Dyadic coping and dyadic adjustment in couples with women with high depressive symptoms during pregnancy. J. Reprod. Infant Psychol. 36, 504–518. 10.1080/02646838.2018.1490496 30068221

[B3] Amato P. R. Booth A. Johnson D. R. Johnson D. R. Rogers S. J. (2007). Alone Together: How Marriage in America is Changing. Cambridge, MA: Harvard University Press.

[B4] Amiri M. Sadeqi Z. Hoseinpoor M. H. Khosravi A. (2016). Marital satisfaction and its influencing factors in fertile and infertile women. J. Fam. Reproduc. Health 10, 139–145. 28101115 PMC5241358

[B5] Badr H. Carmack C. L. Kashy D. A. Cristofanilli M. Revenson T. A. (2010). Dyadic coping in metastatic breast cancer. Health Psychol. 29, 169–180. 10.1037/a0018165 20230090 PMC3138118

[B6] Badr H. Herbert K. Bonnen M. D. Asper J. A. Wagner T. (2018). Dyadic coping in patients undergoing radiotherapy for head and neck cancer and their spouses. Front. Psychol. 9:1780. 10.3389/fpsyg.2018.01780 30374316 PMC6196240

[B7] Bayley T. M. Slade P. Lashen H. (2009). Relationships between attachment, appraisal, coping and adjustment in men and women experiencing infertility concerns. Hum. Reproduc. 24, 2827–2837. 10.1093/humrep/dep235 19666931

[B8] Berghuis J. P. Stanton A. L. (2002). Adjustment to a dyadic stressor: a longitudinal study of coping and depressive symptoms in infertile couples over an insemination attempt. J. Consult. Clin. Psychol. 70, 433–438. 10.1037/0022-006X.70.2.433 11952202

[B9] Bodenmann G. (1995). A systemic-transactional view of stress and coping in couples. Swiss J. Psychol. 54, 34–49.

[B10] Bodenmann G. (2000). Stress Und Coping Bei Paaren [Stress and Coping in Couples]. Göttingen: Hogrefe.

[B11] Bodenmann G. (2005). Dyadic coping and its significance for marital functioning, in Couples Coping With Stress: Emerging Perspectives on Dyadic Coping, eds Revenson K. Kayser T. A. Bodenmann G. (Washington, DC: American Psychological Association), 33–49. 10.1037/11031-002

[B12] Bodenmann G. (2008). Dyadisches Coping Inventar: Testmanual [Dyadic Coping Inventory: Test manual]. Bern: Huber.

[B13] Bodenmann G. Cina A. (2005). Stress and coping among stable-satisfied, stable-distressed and separated/divorced Swiss couples: a 5-years prospective longitudinal study. J. Divorce Remarr. 44, 71–89. 10.1300/J087v44n01_04

[B14] Bodenmann G. Meuwly N. Germann J. Nussbeck F. W. Heinrichs M. Bradbury T. N. (2015). Effects of stress on the social support provided by men and women in intimate relationships. Psychol. Sci. 26, 1584–1594. 10.1177/0956797615594616 26341561

[B15] Bodenmann G. Pihet S. Kayser K. (2006). The relationship between dyadic coping and marital quality: a 2-year longitudinal study. J. Fam. Psychol. 20, 485–493. 10.1037/0893-3200.20.3.485 16938007

[B16] Bodenmann G. Plancherel B. Beach S. R. Widmer K. Gabriel B. Meuwly N.. (2008). Effects of coping-oriented couples therapy on depression: a randomized clinical trial. J. Consult. Clin. Psychol. 76:944. 10.1037/a0013467 19045963

[B17] Bodenmann G. Randall A. K. Falconier M. K. (eds.). (2016). Coping in couples: The Systemic Transactional Model (STM), in Couples Coping With Stress: A Cross-Cultural Perspective, (New York, NY: Routledge), 2–22.

[B18] Brandes M. Van Der Steen J. O. M. Bokdam S. B. Hamilton C. J. C. M. De Bruin J. P. Nelen W. L. D. M.. (2009). When and why do subfertile couples discontinue their fertility care? A longitudinal cohort study in a secondary care subfertility population. Hum. Reproduc. 24, 3127–3135. 10.1093/humrep/dep340 19783833

[B19] Breitenstein C. J. Milek A. Nussbeck F. W. Davila L. Bodenmann G. (2018). Stress, dyadic coping, and relationship satisfaction in late adolescent couples. J. Soc. Pers. Relat. 35, 770–790. 10.1177/0265407517698049

[B20] Chaves C. Canavarro M. C. Moura-Ramos M. (2018). The role of dyadic coping on the marital and emotional adjustment of couples with infertility. Fam. Process. 10.1111/famp.12364. [Epub ahead of print] 29709057

[B21] Cigoli V. Scabini E. (2006). Family Identity: Ties, Symbols and Transitions. New York, NY: Taylor.

[B22] Davidovà K. Pechovà O. (2014). Infertility and assisted reproduction technologies through a gender lens. Hum. Affairs 24, 363–375. 10.2478/s13374-014-0234-9

[B23] Donato S. Iafrate R. Barni D. Bertoni A. Bodenmann G. Gagliardi S. (2009). Measuring dyadic coping: The factorial structure of Bodenmann's “Dyadic Coping Questionnaire” in an Italian sample. TPM 16, 25–47.

[B24] Donato S. Parise M. Iafrate R. Bertoni A. Finkenauer C. Bodenmann G. (2014). Dyadic coping responses and partners' perceptions for couple satisfaction. An actor–partner interdependence analysis. J. Soc. Pers. Relation. 32, 580–600. 10.1177/0265407514541071

[B25] El Kissi Y. Romdhane A. B. Hidar S. Bannour S. Ayoubi Idrissi K. Khairi H.. (2013). General psychopathology, anxiety, depression and self-esteem in couples undergoing infertility treatment: a comparative study between men and women. Eur. J. Obstetr. Gynecol. Reproduc. Biol. 167, 185–189. 10.1016/j.ejogrb.2012.12.014 23298895

[B26] European IVF-monitoring Consortium (EIM) European Society of Human Reproduction and Embryology (ESHRE) Calhaz-Jorge C. De Geyter C. Kupka M. S. de Mouzon J.. (2017). Assisted reproductive technology in Europe, 2013: results generated from European registers by ESHRE. Hum. Reproduc. 32, 1957–1973. 10.1093/humrep/dex264 29117383

[B27] Falconier M. K. Jackson J. B. Hilpert P. Bodenmann G. (2015). Dyadic coping and relationship satisfaction: a meta-analysis. Clin. Psychol. Rev. 42, 28–46. 10.1016/j.cpr.2015.07.002 26295276

[B28] Ferraretti A. P. Nygren K. Nyboe Andersen A. de Mouzon J. Kupka M. Calhaz-Jorge C. (2017). Trends over 15 years in ART in Europe: an analysis of 6 million cycles. Hum. Reproduc. Open 2 10.1093/hropen/hox012 PMC6276702 31486803

[B29] Gager C. T. Sanchez L. (2003). Two as one? Couples' perceptions of time spent together, marital quality, and the risk of divorce. J. Fam. Issues 24, 21–50. 10.1177/0192513X02238519

[B30] Gameiro S. Boivin J. Peronace L. Verhaak C. M. (2012). Why do patients discontinue fertility treatment? A systematic review of reasons and predictors of discontinuation in fertility treatment. Hum. Reproduc. Update 18, 652–669. 10.1093/humupd/dms031 22869759 PMC3461967

[B31] Gana K. Jakubowska S. (2016). Relationship between infertility-related stress and emotional distress and marital satisfaction. J. Health Psychol. 21, 1043–1054. 10.1177/1359105314544990 25139894

[B32] Gentili P. Contreras L. Cassaniti M. D'Arista F. (2002). La dyadic adjustment scale: una misura dell'adattamento di coppia [The dyadic adjustment scale: A measure of couple adjustment]. Minerva Psichiatr. 43, 107–116.

[B33] Ghafouri S. F. Ghanbari S. Fallahzadeh H. Shokri O. (2016). The relation between marital adjustment and posttraumatic growth in infertile couples: the mediatory role of religious coping strategies. J. Reproduc. Infertil. 17, 221–229. 27921001 PMC5124341

[B34] Greil A. L. Slauson-Blevins K. S. McQuillan J. Lowry M. H. Burch A. R. Shreffler K. M. (2018). Relationship satisfaction among infertile couples: implications of gender and self-identification. J. Fam. Issues 39, 1304–1325. 10.1177/0192513X17699027

[B35] Greil A. L. Slauson-Blevins K. S. Shreffler K. M. Johnson K. M. Lowry M. H. Burch A. R. McQuillan J. (2017). Decline in ethical concerns about reproductive technologies among a representative sample of US women. Public Understand. Sci. 26, 789–805. 10.1177/0963662515625402 26817853 PMC13264266

[B36] Hagedoorn M. Sanderman R. Bolks H. N. Tuinstra J. Coyne J. C. (2008). Distress in couples coping with cancer: a meta-analysis and critical review of role and gender effects. Psychol. Bull. 134, 1–30. 10.1037/0033-2909.134.1.1 18193993

[B37] Helgeson V. S. (2011). Gender, stress and coping, in The Oxford Handbook of Stress, Health and Coping, ed S. Folkman (New York, NY: Oxford University Press), 63–85.

[B38] Hilpert P. Randall A. K. Sorokowski P. Atkins D. D. Sorokowska A. Ahmadi K. (2016). The associations of dyadic coping and relationship satisfaction vary between and within nations: A 35-nation study. Front. Psychol. 7:1106 10.3389/fpsyg.2016.01106 27551269 PMC4976670

[B39] Iafrate R. Bertoni A. Margola D. Cigoli V. Acitelli L. K. (2012). The link between perceptual congruence and couple relationship satisfaction in dyadic coping. Eur. Psychol. 17, 73–82. 10.1027/1016-9040/a000069

[B40] Istat (2017). Natalità e Fecondità Della Popolazione Residente [Birth and Fertility of the Resident Population]. Statistiche report. Roma: Istituto Nazionale di Statistica.

[B41] Jackson J. B. Miller R. B. Oka M. Henry R. G. (2014). Gender differences in marital satisfaction: a meta-analysis. J. Marriage Fam. 76, 105–129. 10.1111/jomf.12077

[B42] Kamp Dush C. M. Taylor M. G. Kroeger R. A. (2008). Marital happiness and psychological well-being across the life course. Fam. Relations 57, 211–226. 10.1111/j.1741-3729.2008.00495.x 23667284 PMC3650717

[B43] Kenny D. A. Kashy D. A. Cook W. L. (2006). Dyadic Data analysis. New York, NY: Cambridge University Press.

[B44] Koert E. Daniluk J. C. (2018). When time runs out: reconciling permanent childlessness after delayed childbearing. J. Reprod. Infant Psychol. 35, 342–352. 10.1080/02646838.2017.1320363 29517370

[B45] Kroemeke A. Kubicka E. (2018). Positive and negative adjustment in couples undergoing infertility treatment: the impact of support exchange. PLoS ONE 13:e0200124. 10.1371/journal.pone.0200124 29953537 PMC6023214

[B46] Landis M. Peter-Wight M. Martin M. Bodenmann G. (2013). Dyadic coping and marital satisfaction of older spouses in long-term marriage. GeroPsych 26, 39–47. 10.1024/1662-9647/a000077

[B47] Ledermann T. Bodenmann G. Cina A. (2007). The efficacy of the Couples Coping Enhancement Training (CCET) in improving relationship quality. J. Soc. Clin. Psychol. 26, 940–959. 10.1521/jscp.2007.26.8.940

[B48] Ledermann T. Bodenmann G. Rudaz M. Bradbury T. N. (2010). Stress, communication, and marital quality in couples. Fam. Relat. 59, 195–206. 10.1111/j.1741-3729.2010.00595.x

[B49] Lee T. Y. Sun G. H. (2000). Psychological response of Chinese infertile husbands and wives. Arch. Androl. 45, 143–148. 10.1080/01485010050193913 11111862

[B50] Leuchtmann L. Bodenmann G. (2018). New perspectives on dynamics of dyadic coping, in When “We” are Stressed. A Dyadic Approach to Coping With Stressful Events, eds. Bertoni A. Donato S. Molgora S. (New York, NY: Nova Science Publisher), 3–14.

[B51] Loghi M. Crialesi R. (2017). La Salute Riproduttiva Della Donna [The Reproductive Health of the Woman]. Roma: Istituto Nazionale di Statistica.

[B52] Lowyck B. Luyten P. Corveleyn J. D'Hooghe T. Buyse E. Demyttenaere K. (2009). Well-being and relationship satisfaction of couples dealing with an *in vitro* fertilization/ intracytoplasmic sperm injection procedure: a multilevel approach on the role of self-criticism, dependency, and romantic attachment. Fertil. Steril. 91, 387–394. 10.1016/j.fertnstert.2007.11.052 18281041

[B53] Maroufizadeh S. Karimi E. Vesali S. Omani Samani R. (2015). Anxiety and depression after failure of assisted reproductive treatment among patients experiencing infertility. Int. J. Gynaecol. Obstetr. 130, 253–256. 10.1016/j.ijgo.2015.03.044 26100348

[B54] Martins M. V. Basto-Pereira M. Pedro J. Peterson B. D. Almeida V. Schmidt L.. (2016). Male psychological adaptation to unsuccessful medically assisted reproduction treatments: a systematic review. Hum. Reprod. Update 22, 466–478. 10.1093/humupd/dmw009 27008894

[B55] Martins M. V. Costa P. Peterson B. D. Costa M. E. Schmidt L. (2014a). Marital stability and repartnering: infertility-related stress trajectories of unsuccessful fertility treatment. Fertil. Steril. 102, 1716–1722. 10.1016/j.fertnstert.2014.09.007 25439808

[B56] Martins M. V. Peterson B. D. Almeida V. Mesquita-Guimarães J. Costa M. E. (2014b). Dyadic dynamics of perceived social support in couples facing infertility. Hum. Reproduc. 29, 83–89. 10.1093/humrep/det403 24218401

[B57] Matud M. P. (2004). Gender differences in stress and coping styles. Pers. Individ. Dif. 37, 1401–1415. 10.1016/j.paid.2004.01.010

[B58] Molgora S. Acquati C. Fenaroli V. Saita E. (2018). Dyadic coping and marital adjustment during pregnancy: a cross-sectional study on Italian couples expecting their first child. Int. J. Psychol. 10.1002/ijop.12476. [Epub ahead of print] 29333743

[B59] Monga M. Alexandrescu B. Katz S. E. Stein M. Ganiats T. (2004). Impact of infertility on quality of life, marital adjustment, and sexual function. Urology 63, 126–130. 10.1016/j.urology.2003.09.015 14751363

[B60] Moura-Ramos M. Gameiro S. Canavarro M. C. Soares I. Almeida-Santos T. (2016). Does infertility history affect the emotional adjustment of couples undergoing assisted reproduction? The mediating role of the importance of parenthood. Br. J. Health Psychol. 21, 302–317. 10.1111/bjhp.12169 27059275 PMC5061027

[B61] Pasch L. A. Sullivan K. T. (2017). Stress and coping in couples facing infertility. Curr. Opin. Psychol. 13, 131–135. 10.1016/j.copsyc.2016.07.004 28813283

[B62] Pasha H. Basirat Z. Esmailzadeh S. Faramarzi M. Adibrad H. (2017). Marital intimacy and predictive factors among infertile women in northern Iran. J. Clin. Diagn. Res. 11, QC13–QC17. 10.7860/JCDR/2017/24972.9935 28658854 PMC5483756

[B63] Péloquin K. Brassard A. Arpin V. Sabourin S. Wright J. (2018). Whose fault is it? Blame predicting psychological adjustment and couple satisfaction in couples seeking fertility treatment. J. Psychosomat. Obstetr. Gynecol. 39, 64–72. 10.1080/0167482X.2017.1289369 28635527

[B64] Peterson B. D. Newton C. R. Rosen K. H. (2003). Examining congruence between partners' perceived infertility-related stress and its relationship to marital adjustment and depression in infertile couples. Fam. Process 42, 59–70. 10.1111/j.1545-5300.2003.00059.x 12698599

[B65] Peterson B. D. Newton C. R. Rosen K. H. Schulman R. S. (2006a). Coping processes of couples experiencing infertility. Fam. Relat. 55, 227–239. 10.1111/j.1741-3729.2006.00372.x

[B66] Peterson B. D. Newton C. R. Rosen K. H. Skaggs G. E. (2006b). Gender differences in how men and women who are referred for IVF cope with infertility stress. Hum. Reproduct. 21, 2443–2449. 10.1093/humrep/del145 16675482

[B67] Peterson B. D. Pirritano M. Block J. M. Schmidt L. (2011). Marital benefit and coping strategies in men and women undergoing unsuccessful fertility treatments over a 5-year period. Fertil. Steril. 95, 1759–1763. 10.1016/j.fertnstert.2011.01.125 21333986

[B68] Peterson B. D. Pirritano M. Christensen U. Schmidt L. (2008). The impact of partner coping in couples experiencing infertility. Hum. Reproduc. 23, 1128–1137. 10.1093/humrep/den067 18325885

[B69] Regan T. W. Lambert S. D. Kelly B. McElduff P. Girgis A. Kayser K.. (2014). Cross-sectional relationships between dyadic coping and anxiety, depression, and relationship satisfaction for patients with prostate cancer and their spouses. Patient Educ. Couns. 96, 120–127. 10.1016/j.pec.2014.04.010 24880791

[B70] Reis S. Xavier M. R. Coelho R. Montenegro N. (2013). Psychological impact of single and multiple courses of assisted reproductive treatments in couples: a comparative study. Eur. J. Obstetr. Gynecol. Reproduc. Biol. 171, 61–66. 10.1016/j.ejogrb.2013.07.034 23928476

[B71] Rockliff H. E. Lightman S. L. Rhidian E. Buchanan H. Gordon U. Vedhara K. (2014). A systematic review of psychosocial factors associated with emotional adjustment in *in vitro* fertilization patients. Hum. Reprod. Update 20, 594–613. 10.1093/humupd/dmu010 24676468

[B72] Rooney K. L. Domar A. D. (2016). The impact of stress on fertility treatment. Curr. Opin. Obstetr. Gynecol. 28, 198–201. 10.1097/GCO.0000000000000261 26907091

[B73] Rottmann N. Hansen D. G. Larsen P. V. Nicolaisen A. Flyger H. Johansen C.. (2015). Dyadic coping within couples dealing with breast cancer: a longitudinal, population-based study. Health Psychol. 34, 486–495. 10.1037/hea0000218 25730611

[B74] Rusu P. P. Bodenmann G. Kayser K. (2018). Cognitive emotion regulation and positive dyadic outcomes in married couples. J. Soc. Personal Relation. 36, 359–376. 10.1177/0265407517751664

[B75] Samadaee-Gelehkolaee K. McCarty B. W. Khalilian A. Hamzehgardeshi Z. Peyvandi S. Elyasi F.. (2016). Factors associated with marital satisfaction in infertile couple: a comprehensive literature review. Glob. J. Health Sci. 8, 96–109. 10.5539/gjhs.v8n5p96 26652079 PMC4877237

[B76] Schaller M. A. Griesinger G. Banz-Jansen C. (2016). Women show a higher level of anxiety during IVF treatment than men and hold different concerns: a cohort study. Arch. Gynecol. Obstet. 293, 1137–1145. 10.1007/s00404-016-4033-x 26884350

[B77] Schmidt L. (2006). Psychosocial burden of infertility and assisted reproduction. Lancet 367, 379–380. 10.1016/S0140-6736(06)68117-8 16458748

[B78] Schmidt L. Holstein B. Christensen U. Boivin J. (2005). Does infertility cause marital benefit? An epidemiological study of 2,250 women and men in fertility treatment. Patient Educ. Counsel. 59, 244–251. 10.1016/j.pec.2005.07.015 16310331

[B79] Schwerdtfeger K. L. Shreffler K. M. (2009). Trauma of pregnancy loss and infertility among mothers and involuntarily childless women in the United States. J. Loss Trauma 14, 211–227. 10.1080/15325020802537468 21686042 PMC3113688

[B80] Shapiro C. (2009). Therapy with infertile heterosexual couples: It's not about gender – or is it? Clin. Soc. Work J. 37, 140–149. 10.1007/s10615-008-0149-1

[B81] Sina M. TerMeulen R. Carrasco de Paula I. (2010). Human infertility: is medical treatment enough? A cross-sectional study of a sample of Italian couples. J. Psychosomat. Obstetr. Gynecol. 31, 158–167. 10.3109/0167482X.2010.487952 20569189

[B82] Spanier G. (1976). Measuring dyadic adjustment: new scales for assessing the quality of marriage and similar dyads. J. Marriage Fam. 38, 15–28. 10.2307/350547

[B83] Staff H. R. Didymus F. F. Backhouse S. H. (2017). The antecedents and outcomes of dyadic coping in close personal relationships: a systematic review and narrative synthesis. Anxiety Stress Coping 30, 498–520. 10.1080/10615806.2017.1329931 28513191

[B84] Stanhiser J. Steiner A. Z. (2018). Psychosocial aspects of fertility and assisted reproductive technology. Obstet. Gynecol. Clin. North Am. 45, 563–574. 10.1016/j.ogc.2018.04.006 30092929

[B85] Switzer A. Caldwell W. da Estrela C. Barker E. T. Gouin J.-P. (2018). Dyadic coping, respiratory sinus arrhythmia, and depressive symptoms among parents of preschool children. Front. Psychol. 9:1959. 10.3389/fpsyg.2018.01959 30386280 PMC6198049

[B86] Tao P. Coates R. Maycock B. (2012). Investigating marital relationship in infertility: a systematic review of quantitative studies. J. Reproduc. Infertil. 13, 71–80. 23926528 PMC3719332

[B87] Traa M. J. De Vries J. Bodenmann G. Den Oudsten B. L. (2015). Dyadic coping and relationship functioning in couples coping with cancer: a systematic review. Br. J. Health Psychol. 20, 85–114. 10.1111/bjhp.12094 24628822

[B88] Turner K. Reynolds-May M. F. Zitek E. M. Tisdale R. L. Carlisle A. B. Westphal L. M. (2013). Stress and anxiety scores in first and repeat IVF cycles: a pilot study. PLoS ONE 8:e63743. 10.1371/journal.pone.0063743 23717472 PMC3662783

[B89] Van Der Merwe E. Greeff A. P. (2015). Infertility related-stress within the marital relationship. Int. J. Sex. Health 27, 522–531. 10.1080/19317611.2015.1067275

[B90] Vilchinsky N. Dekel R. (2018). Cardiac disease-induced PTSD: The need for a dyadic perspective, in When “We” are Stressed. A Dyadic Approach to Coping With Stressful Events, eds Bertoni A. Donato S. Molgora S. (New York, NY: Nova Science Publisher), 109–124.

[B91] Vitale S. G. La Rosa V. L. Rapisarda A. M. C. Lagan,à A. S. (2017). Psychology of infertility and assisted reproductive treatment. The Italian situation. J. Psychosomat. Obstetr. Gynecol. 38, 1–3. 10.1080/0167482X.2016.1244184 27750491

[B92] World Health Organization (1992). Recent Advances in Medically Assisted Conception. Geneva: World Health Organization. 1642014

[B93] Yazdani F. Kazemi A. Fooladi M. M. Samani H. R. O. (2016). The relations between marital quality, social support, social acceptance and coping strategies among the infertile Iranian couples. Eur. J. Obstetr. Gynecol. Reproduct. Biol. 200, 58–62. 10.1016/j.ejogrb.2016.02.034 26972768

[B94] Ying L. Y. Wu L. H. Loke A. Y. (2015). Gender differences in experiences with and adjustments to infertility: a literature review. Int. J. Nurs. Stud. 52, 1640–1652. 10.1016/j.ijnurstu.2015.05.004 26021885

[B95] Zegers-Hochschild F. Adamson G. D. Dyer S. Racowsky C. de Mouzon J. Sokol R.. (2017). The international glossary on infertility and fertility care, 2017. Fertil. Steril. 108, 393–406. 10.1016/j.fertnstert.2017.06.005 28760517

[B96] Zimmermann T. Rauch S.-L. (2018). Dyadic coping in patients with prostate and laryngeal cancer and their partners, in When “we” are stressed. A Dyadic Approach to Coping With Stressful Events, eds Bertoni A. Donato S. Molgora S. (New York, NY: Nova Science Publisher), 125–138.

[B97] Zurlo M. C. Cattaneo Della Volta M. F. Vallone F. (2018). Predictors of quality of life and psychological health in infertile couples: the moderating role of duration of infertility. Qual. Life Res. 27, 945–954. 10.1007/s11136-017-1781-4 29307056

